# Natural and Synthetic Sortase A Substrates Are Processed by *Staphylococcus aureus* via Different Pathways

**DOI:** 10.1021/acs.bioconjchem.2c00012

**Published:** 2022-03-23

**Authors:** Silvie Hansenová Maňásková, Kamran Nazmi, Wim van’t Hof, Alex van Belkum, Wendy E. Kaman, Nathaniel I. Martin, Enno C. I. Veerman, Floris J. Bikker

**Affiliations:** † Department of Oral Biochemistry, Academic Centre for Dentistry Amsterdam, University of Amsterdam and VU University Amsterdam, 1081 LA Amsterdam, The Netherlands; ‡ Department of Radiotherapy, Erasmus MC Cancer Institute, 3015 CE Rotterdam, The Netherlands; § BaseClear, Sylviusweg 74, 2302 BH Leiden, The Netherlands; ∥ Biological Chemistry Group, Institute of Biology Leiden, Leiden University Sylviusweg 72, 2302 BH Leiden, The Netherlands

## Abstract

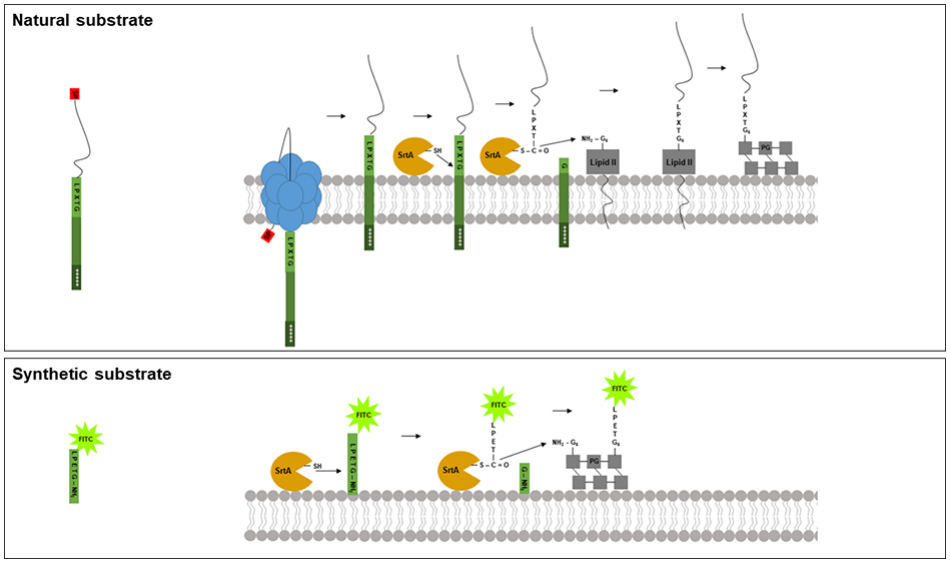

Endogenous *Staphylococcus aureus* sortase A (SrtA) covalently incorporates cell wall anchored proteins equipped with a SrtA recognition motif (LPXTG) via a lipid II-dependent pathway into the staphylococcal peptidoglycan layer. Previously, we found that the endogenous *S. aureus* SrtA is able to recognize and process a variety of exogenously added synthetic SrtA substrates, including K(FITC)LPMTG-amide and K(FITC)-K-vancomycin-LPMTG-amide. These synthetic substrates are covalently incorporated into the bacterial peptidoglycan (PG) of *S. aureus* with varying efficiencies. In this study, we examined if native and synthetic substrates are processed by SrtA via the same pathway. Therefore, the effect of the lipid II inhibiting antibiotic bacitracin on the incorporation of native and synthetic SrtA substrates was assessed. Treatment of *S. aureus* with bacitracin resulted in a decreased incorporation of protein A in the bacterial cell wall, whereas incorporation of exogenous synthetic substrates was increased. These results suggest that natural and exogenous synthetic substrates are processed by *S. aureus* via different pathways.

*Staphylococcus aureus* expresses a large number of virulence factors, which play key roles in successful colonization of a susceptible host as well as establishment of an infection.^1,2^ Among these is a variety of proteins that are covalently linked to the peptidoglycan layer of the bacterium, designated cell wall anchored (CWA) proteins. CWA proteins have been implicated in a variety of processes important for successful colonization and infection, such as adhesion to host tissues, invasion of epithelial cells, evasion of the host’s immune system, and biofilm formation.^1^ Covalent anchoring of these CWA proteins into the staphylococcal cell wall is catalyzed by sortase A (SrtA), a membrane-associated peptidase.^3^ CWA proteins of *S. aureus* share a common architecture in their C-terminal region encompassing three domains: (i) the SrtA recognition motif Leu-Pro-X-Thr-Gly, LPXTG (X represents any possible amino acid); (ii) a hydrophobic transmembrane domain; and (iii) a tail of positively charged amino acids.^4,5^ The last two domains sequester the CWA proteins to the plasma membrane prior to recognition of the LPXTG motif by SrtA. SrtA cleaves LPXTG between Thr and Gly,^6^ resulting in the formation of an acyl-enzyme intermediate. This intermediate is resolved by a nucleophilic attack by the amino group of the pentaglycine side chain of lipid II (undecaprenol-pyrophosphoryl-MurNAc(GlcNAc)-Ala-d-*iso*Glu-Lys(ε-Gly_5_)-d-Ala-d-Ala).^7−9^ Then, a trans-glycosylation reaction follows, in which the sugar subunits (MurNAc-GlcNAc) of lipid II within the CWA-lipid II complexes are polymerized with neighboring sugar subunits of other lipid II and/or CWA-lipid II complexes to generate peptidoglycan (PG) strands.^10,11^ Subsequently, the PG strands are cross-linked in a trans-peptidation reaction, in which a Penicillin Binding Protein (PBP) enzymatically cleaves the bond between the terminal d-Ala-d-Ala of lipid II or CWA-lipid II complexes. This results in the concomitant formation of an amide bond with an accessible pentaglycine of a neighboring strand leading to the stable PG structure.^11^ In this way, CWA proteins are incorporated into the growing PG in a lipid II-dependent manner and ultimately exposed on the mature staphylococcal PG.^8,9,12,13^

The CWA protein incorporation can be inhibited with peptidoglycan synthesis inhibiting antibiotics.^8,14^ These include (i) β-lactams such as penicillin G which mimic the d-Ala-d-Ala motif and thereby inhibit the PBP-catalyzed transpeptidation reaction;^15^ (ii) vancomycin, which blocks transglycosylation and transpeptidation reactions by its specific binding to the sterically accessible d-Ala-d-Ala terminus and its bulky structure;^16^ (iii) bacitracin, which selectively binds and sequesters the C55-pyrophosphate species formed and recycled during lipid II synthesis;^17^ (iv) the lantibiotic nisin which recognizes the pyrophosphate moiety of lipid II by its N-terminal region, which contains A and B lantionine rings.^18^ Its C-terminus (containing C, D, and E rings) is responsible for subsequent pore formation.^19,20^

In previous studies, it was found that, besides the endogenous CWA proteins, SrtA also incorporated exogenously added synthetic SrtA substrates equipped with the SrtA recognition motif.^21−23^ However, only little, if any, competition seemed to occur with natural substrates. Moreover, while incorporation of the natural substrates peaked in the logarithmic growth phase, the highest incorporation of synthetic substrates occurred in the stationary phase.^21^

The aim of the present study was to examine the difference in behavior of natural and synthetic SrtA substrates. To do so, the possible role of lipid II in protein A incorporation, a natural SrtA substrate, was compared with that of two synthetic substrates; K(FITC)LPMTG-amide and K(FITC)-K-vancomycin-LPMTG-amide.^22^

## Results and Discussion

Previous studies showed that exogenous, synthetic SrtA substrates were covalently anchored into the bacterial cell wall by endogenous SrtA.^23^ These studies, however, left the role of lipid II (the primary acceptor for CWA proteins) in this process unresolved.^8,9,14^ To study the role of lipid II in incorporation of native and synthetic SrtA substrates, the effect of several antibiotics targeting different stages of the peptidoglycan synthesis were tested for their effect on SrtA-mediated incorporation of endogenous protein A and exogenously added K(FITC)LPMTG-amide and K(FITC)-K-vancomycin-LPMTG-amide. This was tested using a *S. aureus* 8325-4 WT (wild-type) strain and its isogenic *srtA* deletion mutant (*srtA* KO). The *srtA* KO mutant^21^ was used as a control to confirm the role of SrtA in incorporation of the native and synthetic substrates. The antibiotics included in this study were as follows: penicillin G, vancomycin, and bacitracin. Data on the effects of penicillin G and vancomycin are not shown, as their effects seemed multivariable in pilot experiments including those on the viability on the cells rendering the interpretation of their effects difficult. Bacitracin treatment, however, generated the most reproducible results; therefore, we selected this antibiotic to perform the experiments.

**Figure 1:**
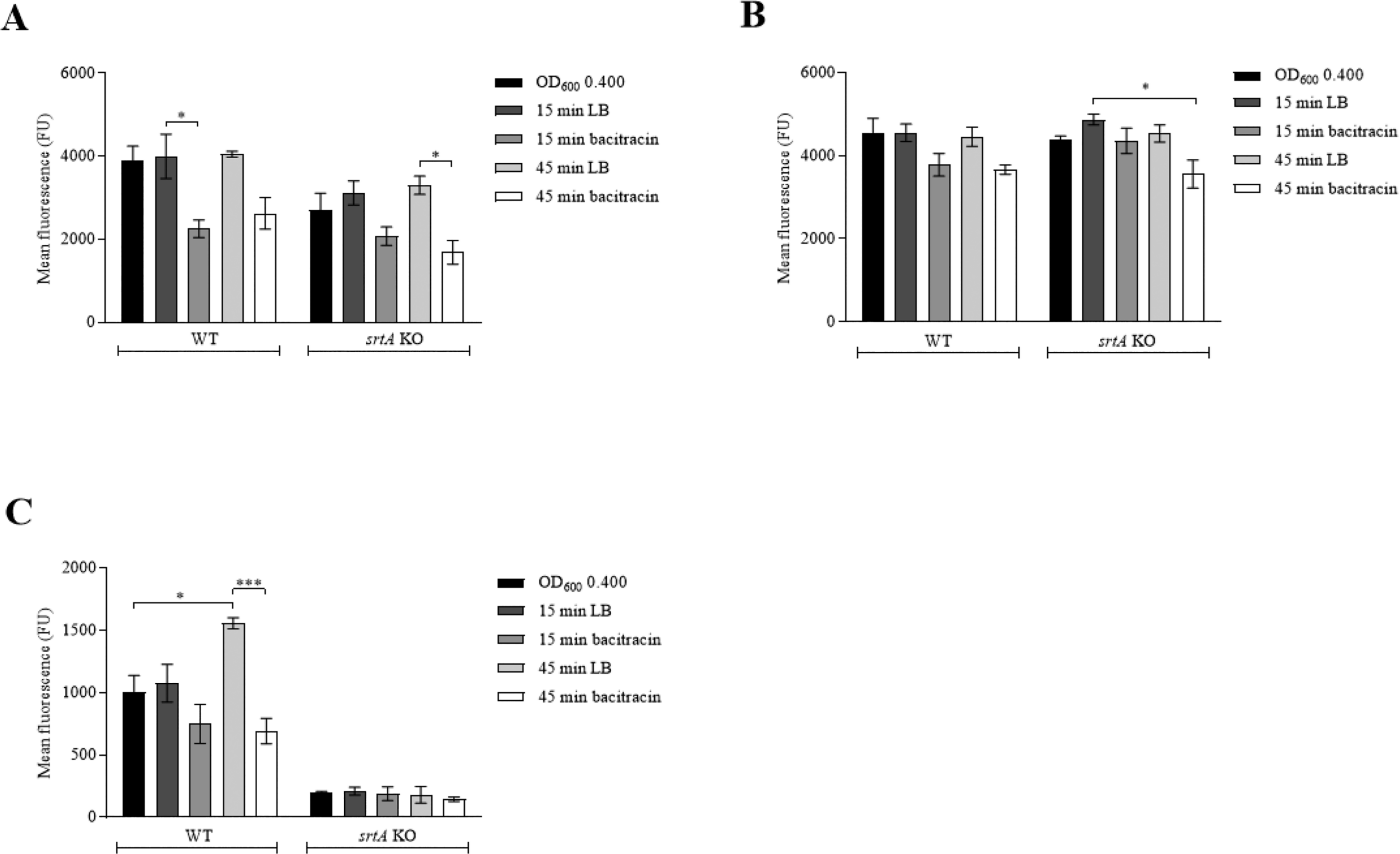
Detection of protein A incorporation and vancomycin and nisin A/B domain binding upon lipid II inhibition with bacitracin. (A–C) WT and *srtA* KO *S. aureus* strains were cultured until OD_600_ 0.400. Then, either LB medium or 1 mg/mL bacitracin was added for either 15 or 45 min. Next, lipid II (A), free d-Ala-d-Ala (B), and protein A (C) presence was determined using FITC-nisin A/B domain, vancomycin–BODIPY conjugate or antiprotein A IgY, respectively. The mean fluorescence (reflecting the binding of the individual reagents to its ligand) was determined by FACS analysis. Significant differences were determined using one-way ANOVA testing with Bonferroni correction. * *P* ≤ 0.05, *** *P* ≤ 0.001.

To limit the pleiotropic effects of bacitracin on the physiology of the cell, the bacteria were treated for short periods of time, 15 and 45 min, respectively. These short-term bacitracin pulses had no effect on bacterial growth, as monitored by OD_600_ measurements and by FACS (maximal decrease in bacterial density of 10%, data not shown). Bacterial concentration decreased with 10% at maximum, and no population shifts were observed within the bacitracin treated population (data not shown). The effect of bacitracin on the expression of lipid II was measured by incubating the bacteria afterward with the FITC-nisin A/B domain or BODIPY-labeled vancomycin, binding to the pyrophosphate linkage group and the d-Ala-d-Ala moiety of lipid II, respectively. Bacitracin pulses resulted in a significant reduction of the FITC-nisin A/B domain binding II in both the *S. aureus* WT as the *srtA* KO strain (*P* = 0.01) compared to the LB- and untreated controls (Figure 1A). Only a moderate decrease in vancomycin–BODIPY binding was measured in both *S. aureus* strains (Figure 1B). This discrepancy could be due to an artifact of the detection method, such as a high background signal produced by existing free d-Ala-d-Ala residues in the existing PG. These data are in line with a previous study performed on *Bacillus subtilis*, where it was found that vancomycin binds to the externalized membrane-bound unincorporated lipid II molecules as well as to free d-Ala-d-Ala residues within the peptidoglycan layer.^24^ No differences were observed between the *S. aureus* WT and *srtA* KO strain (Figure 1A and B). These findings support the view that binding of vancomycin and nisin A/B to the bacterial cell wall is independent of SrtA activity.

**Figure 2:**
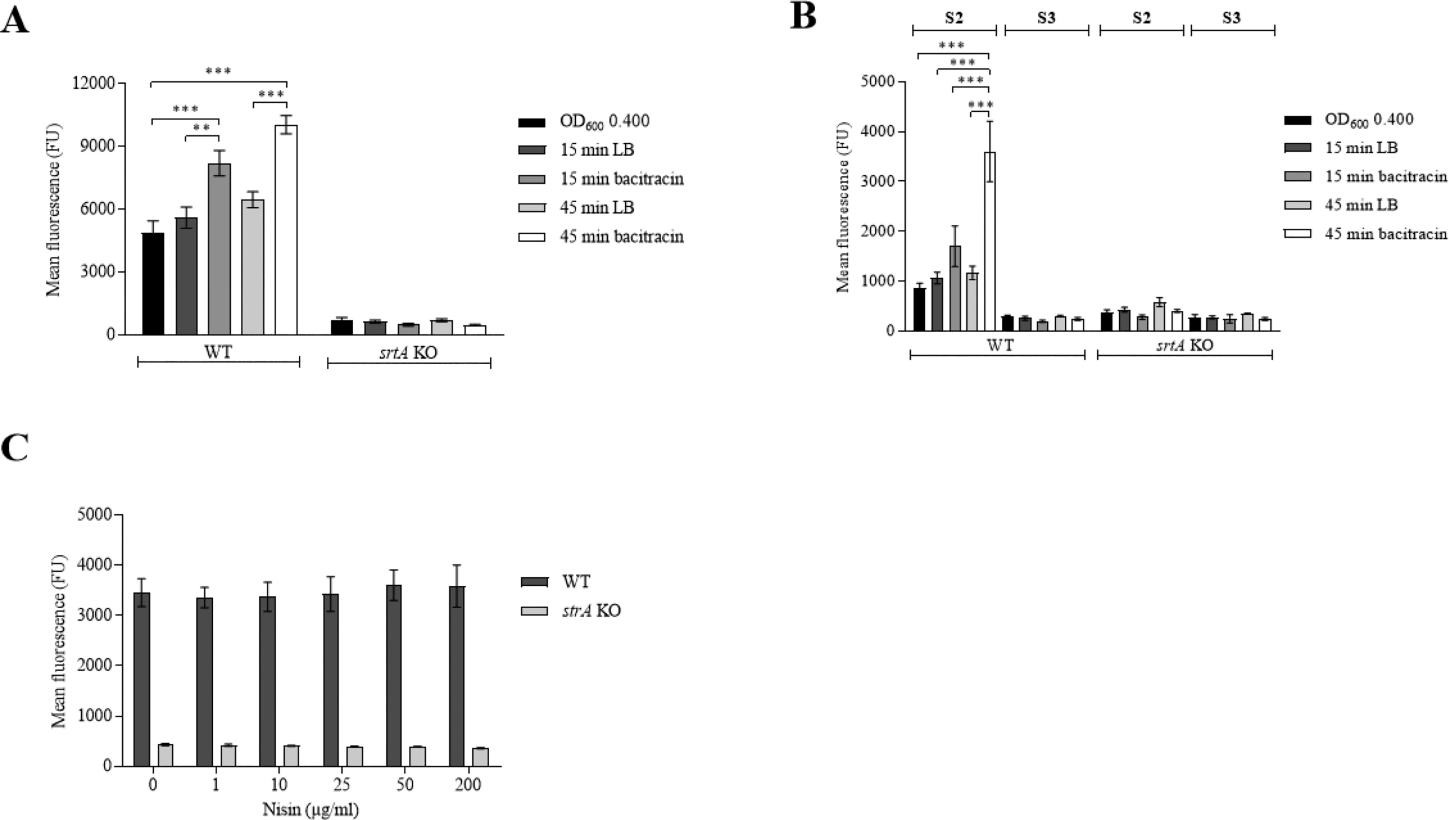
Detection of SrtA synthetic substrate incorporation after lipid II inhibition with bacitracin or nisin A/B domain. (A,B) WT and *srtA* KO *S. aureus* strains were cultured in the presence of either LB medium or bacitracin, as depicted next to the figures and as described in the legend of Figure 1 in more detail. Then, the bacteria were incubated with either 1 mM of substrate 1 (S1 = K(FITC)LPMTG-amide) (A), 5 μM of substrate 2 (S2 = K(FITC)-K-vancomycin-LPMTG-amide) (B), or 5 μM of substrate 3 (S3 = K(FITC)-K-vancomycin-MGTLP-amide) (B) in SrtA buffer. (C) WT and *srtA* KO *S. aureus* bacteria were incubated with increasing concentrations of nisin A/B domain (0–200 μg/mL, depicted on the *x*-axis) in SrtA buffer. Then, bacteria were incubated with 1 mM of substrate **1** (S1 = K(FITC)LPMTG-amide), and mean fluorescence was determined using FACS analysis. Significant differences were determined using one-way ANOVA testing with Bonferroni correction. ** *P* ≤ 0.01, *** *P* ≤ 0.001.

FACS analysis revealed an approximately 1.5-fold (*P* = 0.01) increase in the protein A content in untreated bacteria between 15 and 45 min of culturing (Figure 1C). In the presence of bacitracin, however, protein A was no longer incorporated in this time interval and resulted in a significantly lower concentration (*P* = 0.0001) of protein A after 45 min of bacitracin treatment (Figure 1C). These results support previous studies showing that protein A is anchored in the peptidoglycan layer via a lipid II-dependent pathway.^8^ In parallel, the effect of bacitracin treatment was tested on incorporation of K(FITC)LPMTG-amide and K(FITC)-K-vancomycin-LPMTG-amide. By using these synthetic SrtA substrates, an enhanced incorporation was observed in the bacitracin-treated bacteria (Figure 2A,B).

After growing the bacteria for 15 and 45 min in the presence of bacitracin, the incorporation of K(FITC)LPMTG-amide increased approximately 1.5-fold (*P* = 0.0001) and 2-fold (*P* = 0.0001), respectively, compared to untreated bacteria (Figure 2A). With the substrate K(FITC)-K-vancomycin-LPMTG-amide, an even higher incorporation was found at 2-fold and 4-fold (*P* = 0.0001) higher than the controls after treatment for 15 or 45 min, respectively (Figure 2B). No incorporation of the scrambled substrate (K(FITC)-K-vancomycin-MGTLP-amide) nor for the synthetic substrates (K(FITC)LPMTG-amide and K(FITC)-K-vancomycin-LPMTG-amide) in the *srtA* KO strain was detected.^21^ This confirms that the incorporation depends solely on staphylococcal endogenous SrtA transpeptidase activity (Figure 2A,B). The significantly higher incorporation of K(FITC)-K-vancomycin-LPMTG-amide supports that vancomycin binds to d-Ala-d-Ala motifs present in the mature peptidoglycan layer (Figure 1B). In addition, this suggests that binding of vancomycin to d-Ala-d-Ala within the sortase substrate increased transpeptidation by SrtA. Furthermore, the increased incorporation of specific SrtA synthetic substrates upon inhibition of lipid II suggests that lipid II does not seem to play a role in the incorporation of these substrates. We additionally examined this by testing the effect of treatment with nisin A/B domain, which binds to the pyrophosphate moiety of lipid II, on the incorporation of K(FITC)LPMTG-amide. This revealed that the nisin A/B domain in concentrations up to 200 μg/mL had no effect on incorporation of this substrate (Figure 2C). This further corroborates that these exogenous SrtA substrates are probably directly covalently linked to free pentaglycines within the mature bacterial cell wall, without intermediate binding to lipid II. The increase of accessible free pentaglycine units within the staphylococcal PG can be explained by the decreased availability of CWA-lipid II adducts after bacitracin treatment, that are covalently linked by PBPs to free pentaglycines within mature PG via a trans-peptidation reaction.^25^ This hypothesis is supported by the data presented in Figures 1C and 2A,B, where we showed that decreased protein A display on the bacterial surface is complementary to the increased SrtA substrate incorporation after bacitracin treatment. In addition, these data suggest that there might be a fraction of SrtA transpeptidase that is untethered, which might be able to utilize the available pentaglycine units within the mature peptidoglycan for incorporation of exogenous synthetic SrtA substrates.

The difference in incorporation pathway might be related to the fact that the production and subsequently coupling of natural cell wall associated proteins by SrtA occurs in an ATP-dependent manner. Additionally, a well-structured cell wall is of utmost importance for bacterial survival. Therefore, it is important that these proteins are coupled to the bacterial cell wall via a solid pathway. The synthetic substrates are present in the external microenvironment of the bacterium and freely accessible to SrtA cleavage. This difference in origin and position might thus be the reason that synthetic substrates are incorporated in the *S. aureus* cell wall without interference of lipid II.

## Conclusion

We have examined whether native and synthetic LPXTG containing substrates are processed by *S. aureus* via the same pathway. Therefore, the effect of the lipid II inhibiting antibiotic bacitracin on the incorporation of native and synthetic SrtA substrates was assessed. It was found that inhibition of lipid II led to a decreased incorporation of the native SrtA substrate protein A in the bacterial cell wall, whereas incorporation of exogenous synthetic substrates was increased. The results of this study suggest that natural and exogenous synthetic SrtA substrates are processed by *S. aureus* via different pathways.
