# Supplementary material for: Natural and Synthetic Sortase A Substrates Are Processed by Staphylococcus aureus via Different Pathways
Source: Bioconjug Chem. 2022 Mar 23;33(4):555–9. doi: 10.1021/acs.bioconjchem.2c00012 (PMC9026250; doi:10.1021/acs.bioconjchem.2c00012)
Supplement: Supplementary file 1 — bc2c00012_si_001.pdf [file bc2c00012_si_001.pdf]

SI 1. MATERIALS AND METHODS

Peptide synthesis and procedure

FITC-labelled peptide synthesis, purification and confirmation of authenticity were performed as previously described [1-3]. The substrates numbered **1-3** used in this study are shown in Table 1. The nisin A/B azide species was prepared as previously described [4]. The unique carboxyl groups of nisin A/B were labelled with 5-(aminoacetamido)fluorescein(AAA-flu) as described by Haser et al [5].

Table 1: The amino acid sequences of the synthetic SrtA substrates used in this study.

Substrate	N-term	Substrate sequence	C-term
**S1**	K(FITC)	LPMTG	amide
**S2**	K(FITC)	K(Vancomycin)LPMTG	amide
**S3**	K(FITC)	K(Vancomycin)MGTLP	amide

Bacterial strains

S. aureus 8325-4 wild type (WT) strain and its isogenic *srtA* deletion mutant (*srtA* KO) were used. The *srtA* KO mutant was generated by *srtA:ermC* allele transduction, as previously described [1]. Mutants were selected and maintained on erythromycin plates (3 µg/ml) [1, 6]. No complemented mutant was used so polar effects cannot be excluded.

Short treatment of *S. aureus* with bacitracin

WT and *srtA* KO strains were grown overnight on brain-heart infusion agar (BHA, BD (Becton Dickinson and Company)-Difco, Etten-Leur, The Netherlands) and BHA supplemented with 3 µg/ml erythromycin (Abbott Laboratories, U.S.), respectively. Then, the bacteria were suspended in a volume of 4 ml in Luria-Bertani (LB) medium (BD-Difco) to obtain an OD_{600nm} of 0.400. One tube (time-point zero) was stored on ice until measurement. To the other tubes either LB medium or 1 mg/ml bacitracin (Sigma Aldrich, 50 µl per tube) was added and incubated for either 15 or 45 min. Then, bacteria were collected by centrifugation (5 min at 3,700 x *g*) and suspended in PBS to an OD_{600nm} of 0.400. The bacteria were washed twice with PBS followed by centrifugation for 5 min at 3,700 x *g*. One half of the individual bacterial pellets was suspended in 50 µl PBS and the other half was suspended in SrtA buffer. Ten microliter of individual bacterial pellet per strain per condition suspended in PBS was incubated with either 10 µl of 200 µg/ml FITC-labelled nisin A/B domain, 10 µl of 1 µg/ml vancomycin-BODIPY (4,4-difluoro-4-bora-3a,4a-diaza-*s*-indacene) conjugate (Invitrogen) or 10 µl of 1:50 diluted anti-protein A IgY for 45 min in round bottom plates in the dark at ambient temperature with continuous shaking (800 rpm). In parallel, 25 µl of individual bacterial pellets per strain per condition suspended in SrtA buffer were incubated with 25 µl of either K(FITC)LPMTG-amide substrate (final concentration 1 mM), K(FITC)-K-vancomycin-LPMTG-amide or K(FITC)-K-vancomycin-MGTLP-amide substrates (both final concentration 5 µM) in a flat bottom plates for 17 hrs in the dark at 37 ^{0}C with continuous shaking (220 rpm). Additionally the effect of nisin A/B domain treatment was determined. In brief, WT and *srtA* KO bacteria were cultured until the exponential growth phase reached an OD_{600} of 0.5 at 37 ^{0}C with continuous shaking (230 rpm). After incubation, bacteria were washed twice with PBS followed by centrifugation for 5 min at 3,700 x *g*. The pellet was suspended in 100 µl SrtA buffer and 0, 1, 10, 25, 50 or 200 µg/ml nisin A/B domain was added. The individual cultures were incubated

for 45 min at ambient temperature with continuous shaking (150 rpm). After incubation with nisin A/B domain bacteria were incubated with 1 mM of K(FITC)LPMTG-amide overnight in 50 µl (25 µl bacterial culture in SrtA buffer and 25 µl substrate dissolved in SrtA buffer). Then, the bacteria were washed three times with PBS followed by centrifugation for 5 min at 3,700 x *g*. The bacteria incubated with the synthetic SrtA substrates were treated with 1 % SDS at 60 ^{0}C for 5 min to remove the non-covalently bound and intracellular substrate. Upon three additional PBS washes the stained bacteria were suspended in 200 µl PBS and analysed using the Accuri C6 Flow Cytometer with Accuri C6 software (BD Biosciences).

Statistical analysis

Statistical analysis was performed using the Prism 5.0 package (GraphPad software, San Diego, CA, USA). One-way ANOVA testing with Bonferroni correction was used considering $P < 0.05$ as being statistically significant. Results were depicted as the mean of three independent experiments with Standard Error of the Mean (SEM).

References

(1) Hansenova Manaskova, S.; Nazmi, K.; van Belkum, A.; Bikker, F.J.; van Wamel, W.J. et al. Synthetic LPETG-containing peptide incorporation in the Staphylococcus aureus cell-wall in a sortase A- and growth phase-dependent manner. PLoS One 2014, 9, e89260.

(2) Popp, M.W.; Antos, J.M.; Ploegh, H.L. Site-specific protein labeling via sortase-mediated transpeptidation. Curr Protoc Protein Sci 2009, Chapter 15, Unit 15 13.

(3) Bolscher, J.G.; Oudhoff, M.J.; Nazmi, K.; Antos, J.M.; Guimaraes, C.P. et al. Sortase A as a tool for high-yield histatin cyclization. FASEB J 2011, 25, 2650-2658.

(4) Koopmans, T.; Wood, T.M.; `t Hart, P.; Kleijn, L.H.; Hendrickx, A.P. et al. Semisynthetic Lipopeptides Derived from Nisin Display Antibacterial Activity and Lipid II Binding on Par with That of the Parent Compound. J Am Chem Soc 2015, 137, 9382-9389.

(5) Hasper, H.E.; Kramer, N.E.; Smith, J.L.; Hillman, J.D.; Zachariah, C. et al. An alternative bactericidal mechanism of action for lantibiotic peptides that target lipid II. Science 2006, 313, 1636-1637.

(6) Mazmanian, S.K.; Liu, G.; Jensen, E.R.; Lenoy, E.; Schneewind, O. Staphylococcus aureus sortase mutants defective in the display of surface proteins and in the pathogenesis of animal infections. Proc Natl Acad Sci U S A 2000, 97, 5510-5515.
